# Current Progress of Ratiometric Fluorescence Sensors Based on Carbon Dots in Foodborne Contaminant Detection

**DOI:** 10.3390/bios13020233

**Published:** 2023-02-07

**Authors:** Jialu Zhang, Huinan Chen, Kaidi Xu, Dongmei Deng, Qixian Zhang, Liqiang Luo

**Affiliations:** 1 School of Medicine, Shanghai University, Shanghai 200444, China; 2 College of Sciences, Shanghai University, Shanghai 200444, China; 3 School of Materials Science and Engineering, Shanghai University, Shanghai 200436, China; 4 Shaoxing Institute of Technology, Shanghai University, Shaoxing 312000, China

**Keywords:** carbon dots, ratiometric fluorescence sensors, foodborne contaminants, application

## Abstract

Carbon dots (CDs) are widely used in the detection of foodborne contaminants because of their biocompatibility, photoluminescence stability, and ease of chemical modification. In order to solve the interference problem of complexity in food matrices, the development of ratiometric fluorescence sensors shows great prospects. In this review, the progress of ratiometric fluorescence sensors based on CDs in foodborne contaminant detection in recent years will be summarized, focusing on the functionalized modification of CDs, the fluorescence sensing mechanism, the types of ratiometric fluorescence sensors, and the application of portable devices. In addition, the outlook on the development of the field will be presented, with the development of smartphone applications and related software helping to better enable the on-site detection of foodborne contaminants to ensure food safety and human health.

## 1. Introduction

Food safety is an important livelihood issue, which is related to human health and social stability. In the past, due to the neglect of food-related contaminants, a large number of contaminants have accumulated in crops and livestock through rivers and lands, eventually entering the human body and causing serious diseases. Foodborne contaminants, which include heavy metals, excessive pesticide residues, adulterated foods, and poor food hygiene, are some of the most serious dangers to public health and place a significant financial and medical burden on individuals [1,2]. Therefore, the analytical testing of food is a key technology for ensuring food safety. A variety of well-established analytical techniques have been applied, such as mass spectrometry, chromatography, electrochemistry, surface plasmon resonance, and enzyme-linked immunosorbent assays [3]. However, the complex equipment requirements, complicated pre-treatment processes, and need for specialized operators make them unsuitable for rapid screening and assays of large numbers of samples in relatively backward areas [2]. Therefore, a quick, sensitive, low-cost, high-throughput analytical approach is urgently required for the detection of pollutants that are present in food [3].

Compared with the conventional approaches, fluorescence biosensors based on nanomaterials have high sensitivity and are easy to operate, meaning they can be used for the detection of various targets in food matrices. Organic fluorescent dyes, metal nanoclusters, quantum dots, and rare earth upconversion nanomaterials are classical fluorescent nanomaterials, but their drawbacks such as their toxicity and photobleaching properties limit their practical applications in food analyses [4,5]. With a particle size of less than 10 nm, carbon dots (CDs) are a novel type of nanomaterial. In 2004, Xu et al. first discovered a luminescent carbon nanoparticle via electro-osmosis, but it was not noticed because of its very low quantum yield [6]. In 2006, Sun et al. synthesized blue-emitting carbon nanoparticles with a diameter of less than 10 nm, and officially named the carbon nanoparticles “carbon dots” [7]. According to the quantum confinement and crystal structure, CDs can be classified into graphene quantum dots (GQDs), carbon quantum dots (CQDs), carbon nanodots, and polymer dots [8]. Numerous functional groups that CDs have on their surfaces provide them with exceptional chemical qualities, such as water solubility, photostability, and biocompatibility. Meanwhile, CDs with better performance can be prepared by doping atoms or using group modifications [9]. CDs are environmentally friendly nanomaterials due to their attractive advantages in fluorescence sensing, and responses to analytes can be achieved using various mechanisms, such as via the static quenching effect (SQE), fluorescence resonance energy transfer (FRET), aggregation-induced luminescence (AIE), and the internal filtration effect (IFE) [10]. An increasing number of studies have focused on fluorescent probes for CDs in foodborne contaminants, including pesticides, veterinary medications, bacteria, and prohibited additives, indicating promising applications in food analyses [11].

The complexity of the matrix effects of food samples can create serious interferences during analyses, posing significant challenges for sensor technologies based on CDs. Currently, the reduced matrix interference and the enrichment of target analytes are suggested as two critical aspects to accomplish sensitive and accurate foodborne contaminant detection [2]. Single-emission fluorescence sensors typically have low sensitivity and low selectivity, because the fluorescence intensity is affected by factors unrelated to the analyte [5]. Nowadays, various efforts have been conducted toward the use of CD sensing platforms for food safety by enhancing the sensitivity, specificity, and interference resistance of the sensors through the use of ligands, molecular blotting, enzymes, and immunoreactivity. Due to the CDs’ inherent morphology, comparable structure, and composition, however, a number of other factors can also affect the sensing signal [10,11]. Ratiometric strategy-based fluorescence visual analyses usually provide two-color fluorescence emission wavelengths and respond to foodborne contaminants based on their intensity ratios, thereby improving the detection accuracy as well as the dynamic response range [3]. In recent years, ratiometric fluorescence sensing has been considered as an effective strategy for CD sensors to meet the requirements for target analyte specific identification [11].

Some reviews on the synthesis, characteristics, and principles of CDs have been published, but a more detailed overview on the use of ratiometric fluorescence sensors (RFS) for the analysis of foodborne contaminants is still lacking [5,9,10]. It is possible that this is the case due to the extraordinarily complicated food matrix and the fact that the fluorescence principle of CDs has not yet been entirely understood. In this review, we will deal with the functionalization of CDs, fluorescence sensing mechanisms, RFS, and applications in portable devices. Additionally, the current challenges and emerging trends in the application of CD sensors for foodborne contaminant analyses will be discussed.

## 2. Functionalized Modifications

Two categories of CD synthesis methods can be simply classified: “top-down” and “bottom-up”. Some reviews have been very comprehensive [12,13,14]. However, the unmodified CDs generally consist of carbon and oxygen elements, and their poor luminescence efficiency, poor stability, and lack of active sites limit their applications. Therefore, the modification of CDs is a potent tactic to enhance the photophysical and photocatalytic characteristics [8]. CDs with high fluorescence quantum yields (FLQYs), long emission wavelengths, water solubility, and good biocompatibility can be fabricated via functionalization modifications, thereby broadening the scope of CD applications. In the construction of RFS, the modification of CDs is also an essential step to improve the sensitivity and quantum yield. Currently, heteroatom doping and surface functionalization are used as functionalization techniques.

### 2.1. Heteroatom Doping

By incorporating heteroatoms into the carbon skeleton of CDs, heteroatom doping can successfully modify the intrinsic structure and electrical distribution of CDs to enhance their fluorescence properties [15]. This method modulates the electronic properties by changing the Fermi energy level of the CDs, which can provide more active sites on the surface, such as in -OH, -COOH, and -NH_2_, acting as energy traps to improve the fluorescence characteristics [16]. By altering the quantity and type of heteroatom dopants, the CDs’ fluorescence efficiency can be enhanced and their emission peaks can be changed [8]. The main dopants include metal ions (e.g., Mn, Cu, Co, Mg, Gd, Ce, Tb, and Eu) and non-metal dopants (e.g., N, B, S, P, F, and Cl) [16].

Due to the non-metal and carbon atoms’ similar sizes, the electrical structure of the CDs can be altered, leading to homogeneous doping and structural flaws that greatly enhance the CDs’ performance. Gedanken et al. prepared nitrogen-doped CDs (NCDs) with high FLQYs using 4-hydroxybenzaldehyde and 1, 2, 4, 5-benzenetetramine tetrahydrochloride. Due to the chelating functional groups, the as-synthesized NCDs were used to construct a RFS for Mg^2+^ [17]. Deng et al. prepared boron and nitrogen co-doped CQDs using a hydrothermal method using 3-carboxyphenylboronic acid and o-phenylenediamine. Under UV lamp (300 nm) irradiation, the boron and nitrogen co-doped CQD emission bands formed at 356 and 700 nm, and displayed blue-violet emission with a FLQY of 86.58%, constructing a method for the ratiometric fluorescence detection of IO_4_^−^ as well as cell imaging [18].

Metal ions, due to their special chemical characteristics, can be doped with CDs to modulate their energy band structure and optimize their optical properties [16]. Additionally, primarily chelating between metal ions and chemical groups, metal dopants can inhibit the excessive depletion of amino and carboxyl groups in raw CD materials during dehydrate and carbonate [8]. Tan et al. prepared europium-doped CDs (Eu-CDs) via pyrolysis. Based on the enhanced red fluorescence of Eu^3+^ by dipicolinic acid (DPA) through energy transfer, a ratio fluorescence detection method for DPA was established [19].

### 2.2. Surface Modifications

Surface modification is an ideal strategy to functionalize CDs with ions, chemical compounds, polymers, DNA, and proteins [8]. Functional ligands can be easily modified on the surfaces of CDs via specialized interactions such as conjugation, amidation processes, and electrostatic interactions because CDs have an abundance of surface groups [16]. The surface modification of CDs can enhance their solubility and optical properties, which is particularly beneficial for enhancing the FLQY [5,20,21,22,23].

Dong et al. synthesized CDs via the hydrothermal carbonation of m-phenylenediamine and p-aminobenzoic acid, which was then amidated with 11-mercaptoundecanoic acid (MUA) to obtain green fluorescence MUA-CDs with a high FLQY (59.45%). After doxorubicin was mixed with MUA-CDs, the fluorescence of the MUA-CDs decreased at 513 nm and increased at 590 nm based on electrostatic interactions and FRET. Therefore, a ratiometric optical probe with good selectivity, high sensitivity, and excellent interference resistance was constructed for the detection of doxorubicin [20]. Lanthanide metal ions such as Tb^3+^ and Eu^3+^ are often used to modify CDs, which can give CDs some new properties and functions. Feldmann et al. prepared Tb^3+^/Eu^3+^-modified CDs to study the optical properties of CDs modified with lanthanide ions. At a certain excitation wavelength, there was a FRET process between Tb^3+^/Eu^3+^ and CDs with a high QY, which was 85% for Tb-CDs and 75% for Eu-CDs [21]. Therefore, the functionalized modification of CDs can improve their physical and chemical properties, providing more opportunities for the application of RFS. At the same time, functionalized CDs provide rich binding sites for specific bioreceptors, which is crucial for their application as effective biosensing probes and for the photonic immobilization technique [22,23]. 

## 3. Fluorescence Sensing Mechanism

The principle of fluorescence sensors is based on the combination of CDs and analytes, which causes an enhanced or quenched fluorescence signal [5]. Currently, the main response mechanisms based on CDs used in foodborne contaminant detection are the SQE, FRET, AIE, and the IFE [10].

### 3.1. SQE

The SQE’s mechanism involves the interaction of CDs and analytes to form a non-fluorescent ground state matrix. The compound absorbs energy and instantaneously recovers to the ground state without producing photons, resulting in a static burst. The characteristics of the SQE include the following: (1) the fluorescence lifetime does not change; (2) the absorption spectrum of the CDs changes; (3) the increase in temperature affects the stability of the formed ground state complexes [24].

**Figure 1:**
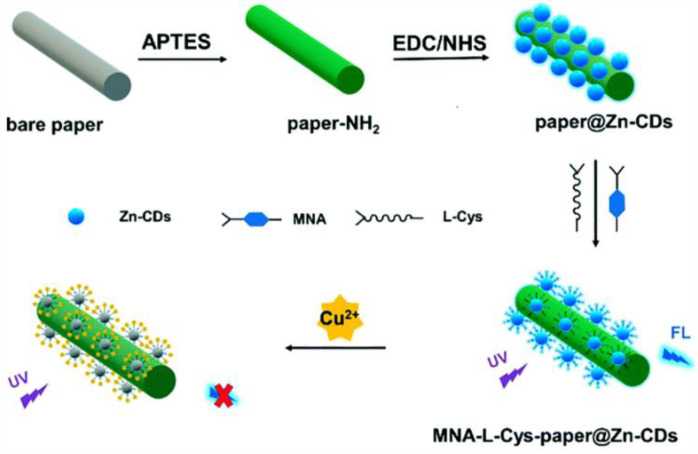
Schematic diagram of the preparation of CDs for the detection of Cu^2+^. Reprinted with permission from [25]. Copyright (2021) Royal Society of Chemistry.

Luo et al. synthesized zinc-doped CDs (Zn-CDs) for the detection of Cu^2+^ by modifying 6-mercaptonicotinic acid (MNA) and L-cysteine (L-Cys) on the surfaces of CDs (Figure 1). It was found that the fluorescence lifetime was unchanged, but the original two absorption peaks of the MNA-L-Cys-Zn-CDs disappeared. Thus, the non-fluorescent ground state complex was formed between MNA-L-Cys-Zn-CDs and Cu^2+^, leading to fluorescence quenching [25]. Similarly, Yao et al. prepared CDs for the rapid detection of Pd^2+^ and Fe^3+^ using bitter melon and a one-step hydrothermal method. According to the research into UV-Vis absorption spectra, fluorescence spectra, and fluorescence lifetimes, the fluorescence mechanism is probably the SQE [26].

### 3.2. FRET

FRET happens between the CDs in the active state and the quencher in the initial state through dipole–dipole interactions when the emission spectra of CDs and the absorption spectra of analytes overlap. Since FRET occurs without the presence of photons, it is a form of non-radiative transfer. The characteristics of FRET include the following: (1) the CDs and quenchers are spaced apart by 1 to 10 nm; (2) the CDs’ fluorescence spectrum and quenchers’ absorption spectra overlap; (3) the CDs’ fluorescence lifetimes are decreased [5,24]. The mechanism of FRET is one of the common methods used to design fluorescence sensors based on CDs, and has been widely used for the detection of foodborne contaminants.

**Figure 2:**
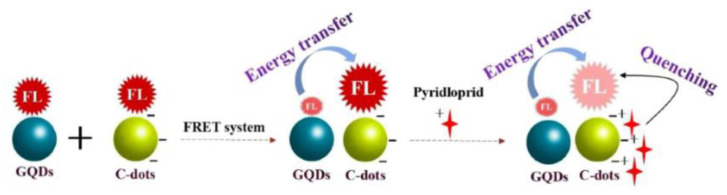
Schematic diagram of the GQD/CD system used for the fluorescence detection of imidacloprid. Reprinted with permission from [27]. Copyright (2022) Elsevier.

Zhang et al. constructed a fluorescence nanoprobe of a GQD/CD FRET system for the detection of imidacloprid by combining GQDs as energy donors and CDs as energy acceptors (Figure 2). The fluorescence of CDs was significantly enhanced due to the effective FRET occurring between GQDs and CDs. When imidacloprid was added to the system, it could hinder the occurrence of FRET, allowing the fluorescence of CDs to be quenched. The constructed fluorescence probes provide a new method for the rapid detection of pesticide residues, with an LOD of 8.23 × 10^−10^ M [27]. Additionally, Pourbasheer et al. constructed a FRET probe for Hg^2+^ detection. In the FRET process, CDs synthesized with citric acid and dimethylglyoxime acted as donors, while silver nanoparticles (AgNPs) acted as acceptors, and the donor–acceptor interaction resulted in the fluorescence quenching of CDs. The fluorescence intensity of the system can be restored via the oxidation of AgNPs by Hg^2+^ ions [28].

### 3.3. IFE

The IFE refers to a non-radiative energy transfer process, which is a phenomenon whereby the excitation or emission spectra of CDs overlap with the excitation spectrum of the analytes, leading to a fluorescence burst of CDs [29]. The features of the IFE consist of the following: (1) it is not a static or dynamic burst process, and does not affect the fluorescence lifetime or absorption peak of the CDs; (2) the fluorescence donor and acceptor distance is not explicitly required. [24]. Thus, the IFE mechanism is simple and flexible. Recently, the prevalence of RFS based on CDs has been increasing [10].

Hu et al. designed RFS based on boron-doped CQDs (B-CQDs) and gold nanoclusters (AuNCs) for the detection of xanthine. Since the UV-Vis absorption spectra of AuNCs and the fluorescence spectrum of B-CQDs effectively coincide, the AuNCs can quench the blue fluorescence of the B-CQDs at the 370 nm excitation wavelength. In addition, no new absorption peaks appeared in the UV-Vis absorption spectra of the system, indicating that no new complexes were formed, and also the fluorescence lifetime did not change. Therefore, the AuNCs quenched the fluorescence of the B-CQDs via the IFE. Then, xanthine oxidase catalyzed the processing of xanthine to produce H_2_O_2_ and uric acid. Under the catalysis of horseradish peroxidase, the generation of •OH from H_2_O_2_ quenched the fluorescence of the AuNCs and restored the fluorescence of the B-CQDs. The fluorescence quenching of the AuNCs was attributed to the strong oxidizing ability of •OH, which can partially solubilize AuNCs and restore the fluorescence of B-CQDs. The RFS showed excellent analytical performance for xanthine, with a detection limit of 0.37 μM, which was applied to the determination of xanthines in human urine [30].

### 3.4. AIE

The discovery of the AIE altered our understanding of the classic effect of aggregation-induced quenching [31]. The AIE is a phenomenon based on the fact that certain fluorophores emit weak fluorescence in dilute solutions; however, the non-radiative energy recession is blocked due to blocked intramolecular motion, and energy can only be released via radiative leaps. Therefore, the intensity increases sharply [32]. The presence of foodborne contaminants may induce the AIE in CDs, which can be used for the control of food and pesticide quality through fluorescence intensity changes [10].

**Figure 3:**
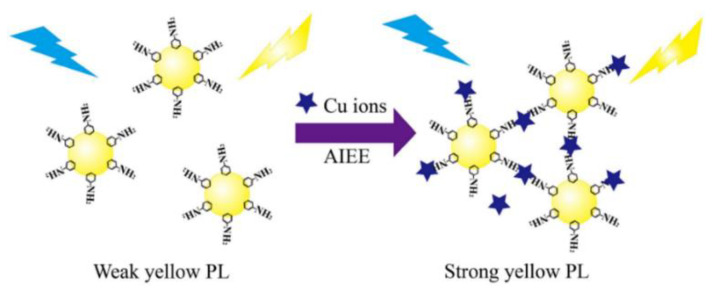
Schematic representation of Cu^2+^ detection by CDs. Reprinted with permission from [34]. Copyright (2019) Elsevier.

Chen et al. prepared CDs with AIE properties via hydrothermal method and constructed a fluorescence sensing system for chlortetracycline (CTC) detection. The hydrogen bonds that formed between the CTC and surfaces of the CDs limited the movement of the CDs and caused them to aggregate, which increased the fluorescence intensity. As a result, a sharp increase in the orange-red fluorescence of the CDs was observed with the addition of CTC. Additionally, the fluorescence intensity reduced and the AIE response of the CD and CTC mixture was dramatically weakened when water was introduced. As a result, the straightforward technique for the dual response to CTC in trace water was established [33]. Huang et al. designed CDs with yellow fluorescence using o-phenylenediamine (OPD) to achieve the detection of Cu^2+^ in the environment and cells (Figure 3). It has been shown that Cu^2+^ induced the aggregation of OPD-CDs, leading to the inhibition of intramolecular vibrations and reducing the non-radiative rate, which enhanced the fluorescence signal of the OPD-CDs [34].

## 4. Ratiometric Fluorescence Probes Based on CDs

The detection of analytes by a single fluorescence signal is inevitably affected by interference from unrelated factors, such as fluctuations in the excitation light source, photobleaching, and light scattering from the sample matrix. Numerous ratiometric quantification methods have been developed and applied to fluorescence sensors for CDs in order to address these issues and improve the sensitivity of the probes. RFS use the ratio of the dual-emission fluorescence signal, which consists of two fluorophores with different emission wavelengths, as the analyte detection signal for quantitative detection. These sensors can be self-calibrated with a detection system for sensitive detection [3,35]. Recently, nanomaterials based on their unique optical properties at the nanoscale have been applied to improve the sensitivity of RFS. In theory, RFS made from nanomaterials can be constructed as long as the fluorescent materials have two or more emission peaks individually or in combination [5,35]. Nowadays, the common fluorescent materials include fluorescent dyes, fluoroproteins, CDs, QDs, noble metal nanoclusters (MNCs), rare earth functionalized nanomaterials, and metal–organic frameworks (MOFs) [3,11,35]. Generally, the RFS based on CDs can be constructed in two forms: (1) CDs with dual-emission properties can be prepared via doping or modification, which can form ratiometric fluorescence probes to detect analytes without other fluorophores or can be applied to pH/temperature sensing and other fields; (2) the RFS can be made of single-emitting CDs and CDs or other fluorophores by changing the simple mixture to be covalent or non-covalent. For foodborne contaminant detection, CDs may be employed as reference or response signals in the RFS [11,35,36]. In this section, we draw conclude the research on RFS made from CDs in foodborne contaminants in the past five years, hoping to inspire relevant researchers in the realm of food detection.

### 4.1. CDs as Dual-Emitting Substrates

When CDs act as dual-emitting substrates, the RFS can be constituted by CDs with dual emissions. The presence of analytes can cause simultaneous quenching of the fluorescence intensities of both emission peaks or the enhancement of one emission peak and the quenching of the other. Accordingly, RFS can be designed based on the ratio of the fluorescence signals [11,35].

The majority of CDs exhibit single-emission fluorescence owing to the similarity of the structures and components of the prepared CDs, and the preparation of multiple-emission CDs still remains challenging. Presently, CDs with multiple emissions have been prepared by coupling fluorophore ligands to the surfaces and intrinsic fluorophores of CDs [11]. The surfaces of multi-emitting CDs may contain multiple functional groups as well as forming multiple surface states to produce multiple emissions under excitation. The intrinsic multiplex emission-based CDs offer potential for designing RFS. Nowadays, they are mostly utilized for heavy metal detection, pH sensing, and cell imaging [37].

Barati et al. prepared novel intrinsic dual-emission CDs for the detection of Cu^2+^ and aspartic acid (Asp) using a one-step hydrothermal method. At the excitation wavelength of 300 nm, CDs can generate double emission peaks at 400 and 610 nm. The emission intensity at 400 nm decreased in the presence of Cu^2+^, while the second emission peak was almost unaffected. The fluorescence emission at 400 nm was progressively restored when Asp was added. Meanwhile, the emission peak at 610 nm was quenched. This RFS provides a new method for the detection of Cu^2+^ in rivers and Asp in human serum samples [38].

Song et al. prepared CDs using a one-step hydrothermal method for the detection of Zn^2+^. Under a single excitation peak (420 nm), the produced CDs displayed three emission peaks of 470, 650, and 685 nm. The addition of metal ions can burst the fluorescence at 685 nm to various degrees. The existence of Zn^2+^ may form CD-Zn^2+^ complexes and can change the surface group distribution of CDs. The complex formation not only effectively quenched the fluorescence at 685 nm, but also significantly enhanced the fluorescence at 650 nm. The uniquely fluorescent response of this nanoprobe was used to analyze Zn^2+^, noticeably enhancing the sensitivity and selectivity of the detection [39].

**Figure 4:**
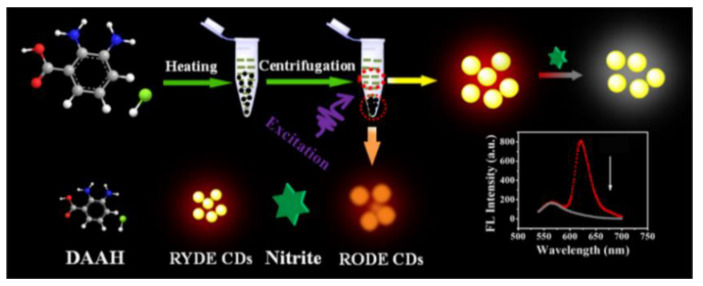
Schematic representation of nitrite detection based on CD ratiometric probes. Reprinted with permission from [40]. Copyright (2019) ACS publications.

The preservative nitrite is frequently used in food, but too much of it can increase the risk of developing cancer and blood pressure problems. Chen and his colleagues prepared the first ratiometric fluorescent nanoprobes with long-wavelength red/yellow dual-emission CDs using a one-pot method (Figure 4). Based on the SQE principle, the probe achieved high sensitivity for the analysis of trace nitrites with LODs of up to 31.61 nM. Moreover, this ratiometric nanoprobe was applied for nitrite detection in bacon, sausages, kimchi, and milk, as well as for the visual monitoring of temperatures and cell imaging [40].

### 4.2. CDs as Reference Signals

The non-fluorescence response of CDs to analytes can be used as a reference signal, while QDs, rare earth functionalized nanomaterials, AuNCs, and organic dyes are often used as response signals constituting ratiometric fluorescence probes [37]. In case the CDs are not completely inert to the analyte, they are often encapsulated into nanoparticles, such as MOFs, silica, and polymer particles, so as to ensure the stability of the signal [36]. Table 1 summarizes the studies on RFS with CDs as reference signals and lists the relevant mechanisms of foodborne contaminant detection.

**Figure 5:**
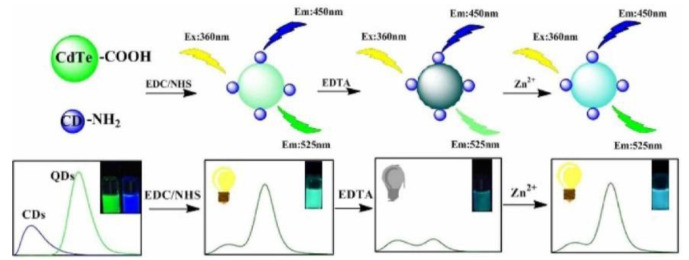
Schematic representation of the synthesis and Zn^2+^ detection of QD/CD ratiometric nanosensors. Reprinted with permission from [41]. Copyright (2018) Elsevier.

QDs have long-wavelength emission behavior. Meanwhile, the size of the QDs can be changed to tailor the energy band. These properties have facilitated the development of unique QD/CD RFS [11]. For instance, Hu et al. created a dual-emission nanoprobe through covalently combining CDs as the reference standard with CdTe QDs as the response signal for Zn^2+^ sensing (Figure 5). The fluorescence of the QDs was quenched after the addition of EDTA, and the QDs fluorescence signal was restored with the increase in Zn^2+^. The probe can be utilized to detect Zn^2+^ with a detection limit of 0.33 μM [41]. Hu et al. used the reference signal of CDs and N-acetyl-L-cysteine-modified CdTe QDs as the response signal for the sensitive detection of CN^−^ in water. The visual monitoring of CN^−^ in aqueous solutions and test strips was achieved using fluorescence probes, and the LOD was as low as 10.35 nM [42]. Hu et al. synthesized CdTe QDs using CDs as capping ligands to prepare ratiometric fluorescence probes for the detection of guanine. In this probe, CDs were introduced as a reference signal by covalent bonds. Therefore, the prepared probe was more stable than the traditional reference fluorescent probe and showed advantages in eliminating the concentration change error [43]. Spermine is an essential indicator for the freshness of meat. Song et al. reported a probe consisting of B-CDs and red fluorescent CdTe QDs, which could be used to visually monitor the freshness of pork. With the addition of spermine, the red fluorescence of the CdTe QDs was gradually quenched, while the fluorescence of the CDs remained unchanged. The probe solution’s fluorescence color changed from light red to blue, meaning the spermine can be specifically and accurately identified from other organic amines with a detection limit of 76 nM [44].

**Figure 6:**
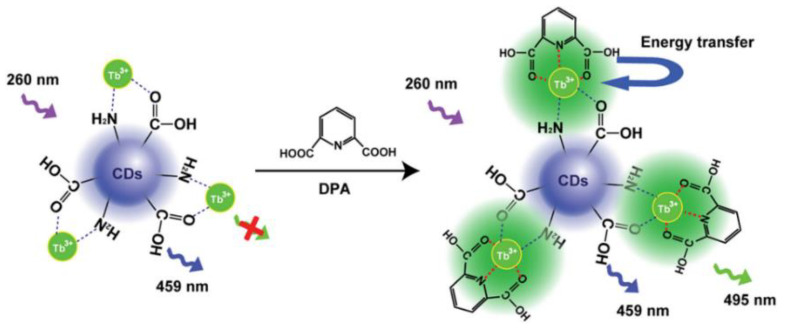
Schematic diagram of the ratiometric fluorescence detection of DPA using CD-Tb. Reprinted with permission from [47]. Copyright (2019) Elsevier.

Rare earth functionalized nanomaterials possess large Stokes shifts, long fluorescence lifetimes, and high photochemical stability [45]. Based on the optical properties of rare earth metals, ratiometric fluorescence probes composed of rare earth metals as response signals and CDs as inner standards are attracting great attention [11]. Tetracycline (TC) is an antibiotic that is frequently prescribed for the treatment of infections in both humans and animals. However, the irrational abuse of TC may seriously undermine human health. Liu et al. designed and synthesized a cyclic structure of CDs ligated with Eu^3+^ to construct a dual-emission sensor for the detection of TC. Upon the addition of TC, a CD-Eu^3+^-TC ternary complex was formed, and its formation caused the Eu^3+^ to emit an intensive red fluorescence. Meanwhile, the blue fluorescence of the CDs remained unchanged and TC detection could be achieved by recording the emission ratio [46]. To the best of our knowledge, DPA is a biomarker of anthrax. Huang and his colleagues designed fluorescent probes of terbium-ion-modified CDs (CDs-Tb) to detect DPA (Figure 6). The addition of DPA affected the Tb^3+^ emission signal enhancement based on the antenna effect (AE), while it had no effect on the emission of CDs. On the other hand, the fluorescence intensity ratio of the CD-Tb probe was directly proportional to the analyte concentration, thereby achieving the sensitive detection of DPA [47]. In addition, Zhang et al. designed CDs chelated Eu-MOFs as ratiometric fluorescence probes to detect the CaDPA. Eu-MOF materials can combine the advantages of Ln^3+^ and MOFs, thereby enabling the sensitive detection of Bacillus anthracis spores [48].

MNCs are widely appreciated for their ease of preparation and surface modification, biocompatibility, and excellent photoluminescence properties. Recently, the dual-emission characteristics afforded by MNC/CDs have created opportunities for ratiometric fluorescent probe design, having been applied to the detection of foodborne contaminants [49]. Joo et al. prepared CDs/AuNCs@ZIF-8 probes by embedding CDs and AuNCs in ZIF-8, where cephalexin quenched the fluorescence of the AuNCs without affecting the signal of the CDs. The probe can be used for the sensitive detection of cephalexin with a low LOD of 0.04 ng/mL [50]. More interestingly, the Gram-negative bacteria could reduce the Cu^2+^ and restore the fluorescence of the AuNCs. Therefore, Jia et al. prepared BCD@SiO_2_@AuNCs nanocomposites as dual-emission fluorescence sensors to allow the more selective detection of Gram-negative bacteria [51].

**Table 1:** Summary of fluorescence sensors with reference signals of the ratiometric CDs.

Fluorescent Probes	Analytes	Detection Mechanism	LOD	Ref.
QDs/CDs	Zn^2+^	Ligand effect	0.33 μM	[41]
QDs/CDs	CN^−^	Ligand effect	10.35 nM	[42]
QDs/CDs	guanine	Excited state electron transfer	3.6 μM	[43]
QDs/CDs	Spermine	Spermine-induced aggregation of CdTe QDs	76 nM	[44]
CDs-Eu^3+^	TC	Energy transfer	11.7 nM	[46]
CDs-Tb	DPA	AE	100 pM	[47]
CDs@Eu-MOFs	CaDPA	AE	0.66 g/L	[48]
CDs/AuNCs@ZIF-8	cephalexin	Complex formation	0.04 ng/mL	[50]
BCD@SiO_2_@AuNC	Gram-negative bacteria	Reduction effect	150 cfu/mL (*E. coli*), 112 cfu/mL (Pseudomonas aeruginosa), 792 cfu/mL (Salmonella typhimurium)	[51]
APTES-NBD-CDs	OA	PET	25 ng/L	[52]
o-CDs@methyl red	Tyramine	pH response	0.01 μM	[53]

Organic dyes are also commonly used as response signals with CDs as internal standards to construct RFS. Yu et al. constructed a dual-emission nanoprobe incorporating molecular blotting with nitrobenzo-oxadiazole and CDs as the response signal and reference signal, respectively. The fluorescence probe can be used for the sensitive detection of okadaic acid in seawater and sediment samples [52]. Biogenic amines (BAs) are crucial markers of food deterioration, and their overconsumption may have a negative impact on human health. Lu et al. prepared a nanoplatform for detecting BAs by covalently binding methyl red on NH_2_-modified CDs. The method, in which methyl red is used as a reporter signal for BAs and the fluorescence signal of o-CDs is uninfluenced, has been successfully applied to the detection of biogenic amines in milk and yogurt samples [53]. Given that the structure of organic dyes is very similar to most detectors, this may affect the fluorescence signal of the CDs and influence the sensitivity of the sensing systems. Consequently, the development of ratiometric fluorescence probes remains highly promising [11].

### 4.3. CDs as Response Signals

As CDs act as fluorescence response signals sensitive to the analyte, another fluorophore can be used as a reference signal to constitute the RFS [37]. In addition, many CD composite fluorescent probes have both signals responding to the analyte. The fluorescence intensity of one signal can be enhanced by the analyte, while that of the other signal is quenched. The ratio of the intensity of the both fluorescence signals is used as a detection index, enabling the ratiometric fluorescence detection of the analyte [35]. In the use of CDs as response signals, the reference signals are generally QDs, MNCs, and Eu^3+^ [37]. Table 2 summarizes the studies of RFS with CDs as response signals and lists the relevant mechanisms for foodborne contaminant detection.

By encapsulating QDs into nanoparticles as internal standards, they can be used with CDs to form ratiometric sensors for foodborne contaminant detection. Compared to QDs, CDs have lower quantum yields and poorer photostability. However, the combination of QDs and CDs can improve the sensitivity and linear range of the probes. Jiang et al. used 3D printing technology to construct a portable platform for pesticide detection, in which CdTe QDs were embedded in SiO_2_NPs as internal standards and B-CDs were attached to the surfaces of SiO_2_NPs as reporter signals. Therein, the AuNPs could quench the blue fluorescence of the bCDs and the fluorescence signal was restored as the addition of fomesin induced the aggregation of AuNPs. Thus, the rQDs@SiO_2_@CD dual-emission ratio fluorescent probe enables the sensitive detection of pesticide residues [54]. Moreover, Xie et al. covalently connected bCDs to the surfaces of silica nanoparticles containing QDs to construct nanoprobes for the detection of Hg^2+^. The QDs in the silica matrix provided a constant reference signal for Hg^2+^ and also improved the optical and chemical stability. However, the bCDs were very sensitive to Hg^2+^, and the probe had high sensitivity and selectivity to Hg^2+^. The technology offers a fresh way to analyze Hg^2+^ and paves the way for real-time pollution control [55].

Among the RFS composed of MNCs and CDs, the fluorescence signal is typically generated according to the FRET mechanism. For example, Fu et al. used CDs as a response signal to design the CDs/CuNCs nanoprobe to detect dopamine (DA) [56]. Wang and his colleagues, based on FRET between N-CQDs and AuNCs, developed a ratiometric fluorescent probe for the responsive detection of polymyxin [57]. Chen et al. constructed a FRET system of AgNPs and CDs for the detection of ochratoxin A in flour and beer. Due to the proximity of CD and AgNPs, the fluorescence intensity of the CDs decreased, while the addition of OTA restored the fluorescence signal of the CDs. The probe has been used to detect ochratoxin A in real agricultural samples with a limit of detection as low as 8.7 nM [58].

**Figure 7:**
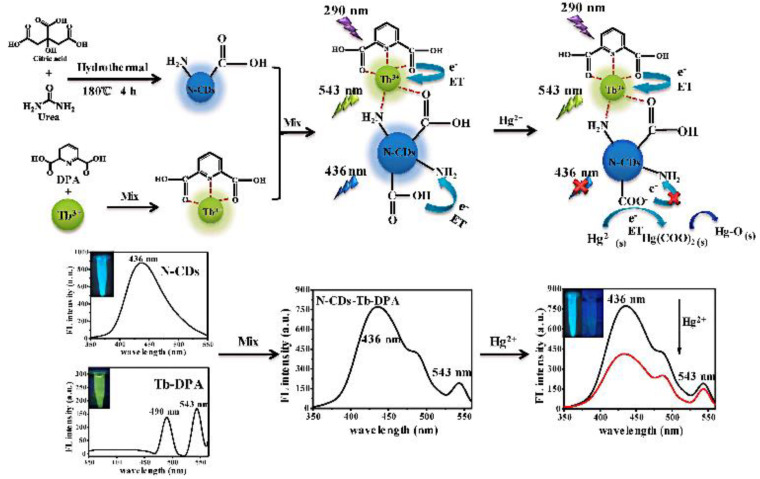
Schematic diagram of the N-CDs-Tb-DPA sensor for Hg^2+^ detection. Reprinted with permission from [60]. Copyright (2020) Elsevier.

**Figure 8:**
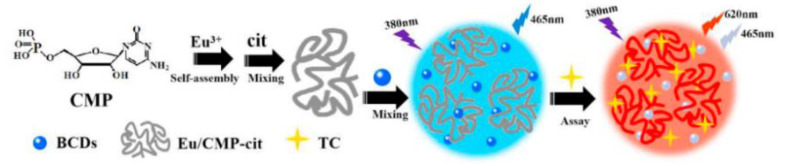
Schematic representation of TC detection based on fluorescent probes for BCDs-Eu/CMP-cit ratio determination. Reprinted with permission from [61]. Copyright (2020) Elsevier.

Lanthanides hold high-performance visible and near-infrared properties. Nowadays, lanthanides can be used as internal standards with CDs as response signals to form ratiometric probes for the detection of metal ions or organic small molecules. Chen et al. prepared ratiometric sensors from CDs with (Gd,Eu)_2_O_3_ rare earth materials using an electrostatic absorption technique. It was demonstrated that curcumin barely influenced the red Eu^3+^ emissions while quenching the blue CDs’ fluorescence. Therefore, a novel fluorescence sensor for curcumin was developed with a detection limit of 0.0615 μg/mL [59]. Li et al. reported on RFS with a combination of Tb-DPA (reference signal) and N-CDs (response signal) for the sensitive detection of Hg^2+^ in seafood [60] (Figure 7). Simultaneously, Xiao et al. designed RFS for TC detection in food by simply mixing bCDs and cytidine monophosphate (CMP)/Eu-coordinated polymer nanoparticles (Eu/CMP-cit CPNs) (Figure 8). With the addition of the TC, the emission of bCDs at 465 nm was diminished, while the emission of Eu/CMP-cit CPNs at 620 nm was enhanced. A probe with an opposite signal response could further increases the sensitivity of the analysis, achieving the detection of trace TC in complex practical samples [61]. Based on a similar principle, Wang et al. prepared a dual-emission fluorescence probe by incorporating Mg,N-CDs in a europium metal–organic framework together with molecular blotting (Mg,N-CDs/Eu-MOFs@MIP) for the detection of oxytetracycline (OTC). Upon the addition of OTC, an efficient sensing mechanism with the synergistic effect of IFE and AE could be triggered to improve the sensitivity of the OTC detection in the milk samples [62].

**Figure 9:**
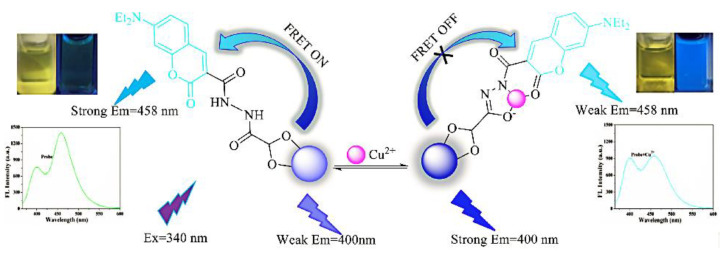
Schematic diagram of CMH-GA-CDs for the detection of Cu^2+^. Reprinted with permission from [65]. Copyright (2018) Elsevier.

Even if organic dyes do not respond to the analyte, they can be bound to CDs in an encapsulated or covalent fashion to form ratiometric fluorescence probes. Liu et al. synthesized the nitrite-sensitive bCDs using a hydrothermal method, and constituted RFS with rhodamine B (RhB) dye. CDs-RhB can be used as a ratiometric probe for the analysis of nitrite in real samples from soil extracts, fish pond water, and so on [63]. Wang et al. constructed a double emission probe for the detection of ciprofloxacin using a simple method of coupling CDs to the fluorescent dye riboflavin [64]. In case the analyte affects the intrinsic emission of both organic dyes and CDs, a dual-emission probe for sensitive detection of the analyte can be constituted. Chen et al. formed a fluorescence probe for the detection of Cu^2+^ by covalently binding 7-diethylamino coumarin-3-carbohydrazide (CMH) to the surface of glyoxylic-acid-modified CDs (GA-CDs) (Figure 9). The principle of CMH-GA-CD probe detection is that the Cu^2+^ coordinates with heteroatoms of N and O on the surface of CDs, thereby inhibiting the FRET between the CDs and CMH. Accordingly, the fluorescence intensity of the CDs is enhanced and that of the CMH is reduced. This method exhibited great accuracy and specificity in Cu^2+^ detection with a limit of detection as low as 0.21 μM [65].

Besides the above-mentioned common fluorescent materials, there are also many fluorophores that can form dual-emitting fluorescence probes with CDs. Lu et al. combined the fluorescence of surface-functionalized CDs with the optical properties of DA polymerization to achieve the detection of organophosphorus pesticides (OPs) based on photoinduced electron transfer. In this sensor design, acetylcholinesterase-mediated DA convergence and the inhibition of the enzyme by OPs are combined. Consequently, the concentration of OPs moderates the ratiometric signal by affecting the degree of DA polymerization. The proposed CDs/DA sensor shows high selectivity for OPs at the pg/L level, providing a new option for the detection of OPs [66]. Lan et al. prepared S,N-CDs using thiourea and citric acid for the detection of TC. The intrinsic fluorescence of TC can be employed as an internal standard, while the addition of TC specifically muted the fluorescence of the CDs. The probe was used to detect residual TC in fish and pork samples [67]. 

### 4.4. Dual CDs

Multiple CDs with single emissions can be simply mixed or coupled with other materials with mesopores to form probes. The RFS based on dual CDs are detected through the use of two different types of CDs for the analyte. One fluorescence CD’s emission peak is employed as the response signal, and the other is used as the reference signal [37]. Dual-fluorescence emission centers can improve the robustness and accuracy during sensor detection through the use of a multidimensional signal output [68].

Pan et al. constructed a double-color CDs ratio nanoprobe to achieve the sensitive detection of alkaline phosphatase (ALP). Blue fluorescent CDs (B-CDs), yellow fluorescent CDs (Y-CDs), and MnO_2_ nanosheets were simply mixed to form B/Y-CDs-MnO_2_ nanocomposites. The probe launched two peaks at 443 nm (B-CDs) and 539 nm (Y-CDs) under excitation at 380 nm, whereas the MnO_2_ nanosheets produced different degrees of quenching of B-CDs and Y-CDs via FRET. The ascorbic acid catalyzed by the ALP was able to reduce the MnO_2_ to Mn^2+^, thereby restoring the fluorescence signal. The ratiometric probe was more capable of detecting ALP, which is crucial for cancer diagnoses and biomedical research [69].

Liu and his colleagues reported on double-emitting CDs with various ligands, which were combined with molecular blotting techniques to achieve the analysis of heavy metals. The presence of the Cr^3+^ only quenched the fluorescence of the CDs, while the Pb^2+^ merely quenched the fluorescence of the CDs. Therefore, this probe can achieve dual-channel detection of Cr^3+^ and Pb^2+^ in parallel. The dual-channel detection technique was used for the analysis of Cr^3+^ and Pb^2+^ in real water samples, and satisfactory recovery results were gained, providing a new method of detection for heavy metal ions in complex water environments [70].

**Figure 10:**
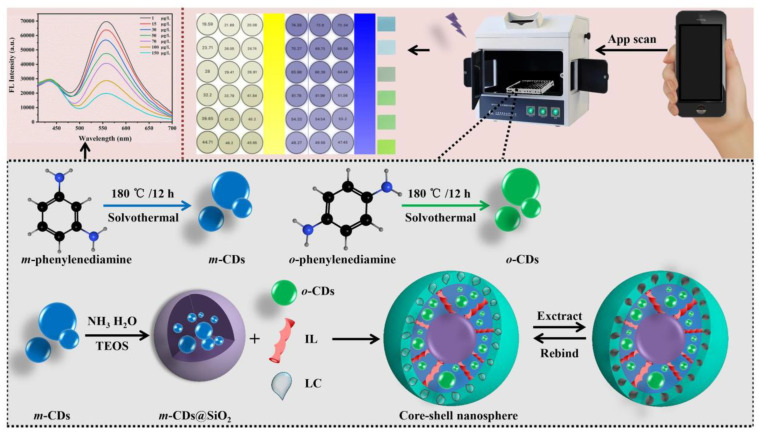
Schematic diagram of scaled core–shell nanospheres for LC detection. Reprinted with permission from [71]. Copyright (2022) Elsevier.

λ-Cyfluthrin (LC), a pyrethroid insecticide, probably has the potential to accumulate in humans and to cause psychiatric diseases. Sun et al. prepared blue-green dual-emitting CDs as optical sensing centers. Subsequently, a novel ratiometric fluorescent core–shell nanosphere was constructed in association with molecular blotting technology to achieve the in situ detection of LC (Figure 10). Under the presence of LC, the blue fluorescence of m-CDs (440 nm) wrapped in SiO_2_ acted as the internal standard and the green fluorescence of o-CDs (550 nm) embedded in the molecularly imprinted polymer layer acted as the response signal. Based on the PET mechanism, the platform combined fluorescence sensing and a smartphone application to achieve the ultra-sensitive detection of LC. Therefore, the sensing platform has great potential for applications in the field detection of pesticide residues and contamination control under resource-limited conditions [71].

The ratiometric fluorescent probe based on CDs effectively avoids the drawbacks of single emissions and achieves the quantitative detection of analytes using the fluorescence intensity ratio of double-emission peaks. For example, Wang et al. constructed an RFS from mesoporous silica doped with CDs and fluorescein loaded in pores for the detection of L-Cys. The sensor effectively eliminates the potential effects of background fluorescence and other analyte-independent external environmental factors using a ratiometric approach. At the same time, the nanosensor was used to monitor the level of L-Cys in human serum, and satisfactory results were achieved compared with single-emission probes [72]. As we know, ratiometric probes generally consist of CDs with fluorescent dyes, metal nanoparticles, or rare earth materials, which are widely used in the detection of foodborne contaminants such as heavy metals, pesticides, antibiotics, and toxins. Meanwhile, these ratiometric probes have great promise in the field of biosensing due to the good biocompatibility of CDs [37].

## 5. The Application of Portable Devices

Food issues are closely related to people’s lives, which is a topic we cannot ignore. Since the detection of foodborne contaminants is time-consuming and labor-intensive, in addition to requiring expensive equipment, detection is difficult in remote areas or places where conditions are more rudimentary. Therefore, the creation of a simple, portable, real-time, and intelligent method of food analysis is critical. Recently, several strategies have been applied and combined with machine learning to develop portable devices, such as visualization test strips, microfluidic paper chips, microarrays, and smartphone technology [11]. Simultaneously, adding detectors in ratiometric fluorescence probes brings about changes in different characteristic tones. The analysis of color tone changes in ratiometric sensing can increase the detection sensitivity, since changes in color are more perceptible to the human eye than changes in luminance [35].

**Figure 11:**
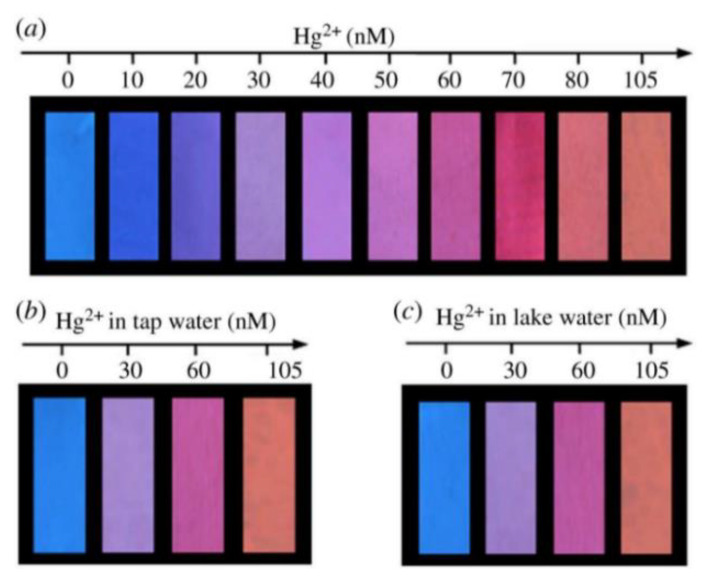
(**a**) The visual detection of Hg^2+^ using fluorescent test paper. (**b**,**c**) The visual detection of Hg^2+^ in tap water and lake water. These photographs were taken under a 365 nm UV lamp. Reprinted with permission from [74]. Copyright (2018) Royal Society of Chemistry.

Paper materials have been widely used in scientific research due to their cheapness, wide availability, and easy modification [73]. By modifying the fluorescent probe on the paper-based material, the material can be solidified and the equipment for the RFS can be simplified. Wang et al. constructed paper-based fluorescence sensors for Hg^2+^ detection by mixing SiNCs with RCDs as fluorescent inks and printing them on filter paper using an inkjet printer. The ratiometric probe used the RCDs’ signal as an internal standard, and the addition of Hg^2+^ quenched the blue fluorescence of the SiNCs, which was visible as a color change to the naked eye under UV light. The constructed paper-based sensor was exposed to various concentrations of Hg^2+^ solution, and the test paper displayed a color shift from blue to orange-red when illuminated by a 365 nm UV light (Figure 11). A unique technique for detecting Hg^2+^ in lake and tap water was provided by the proportional fluorescence test paper [74]. Du et al. prepared ratiometric fluorescent test pens by mixing blue CDs and red CdTe QDs to enable the portable and visual determination of Ag^+^ on a paper base [75].

**Figure 12:**
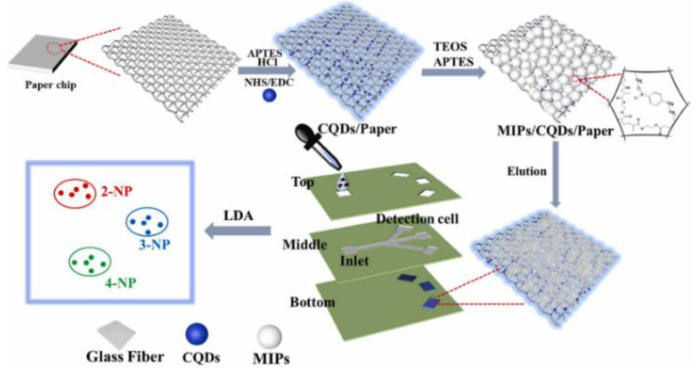
Schematic of the fabrication of the MIP/CQD/μPAD fluorescent sensor array platform. Reprinted with permission from [76]. Copyright (2022) ScienceDirect.

The use of microfluidic paper-based analytical devices (μPADs) with a quick and inexpensive detection technique has received a lot of interest. Li et al. developed a novel fluorescent sensor on microfluidic paper chip combined with molecular blotting for the sensitive and accurate analysis of three nitrobenzyl phenol (NP) isomers in parallel (Figure 12). The blue-emitting CQD was immobilized on glass fiber paper and then the template molecules 2-, 3-, and 4-NP were added, resulting in fluorescence quenching. The removal of template molecules via elution produced recognition sites and restored fluorescent signals. The sensor modules of these fluorescence probes show different binding affinities for NP isomers, resulting in various effects of fluorescence bursts. Therefore, three NPs can be sensitively distinguished by MIP/CQD/μPADs, and μPADs show wide promise as inexpensive and portable devices in the fields of environmental monitoring, disease prognosis, and food safety [76].

**Figure 13:**
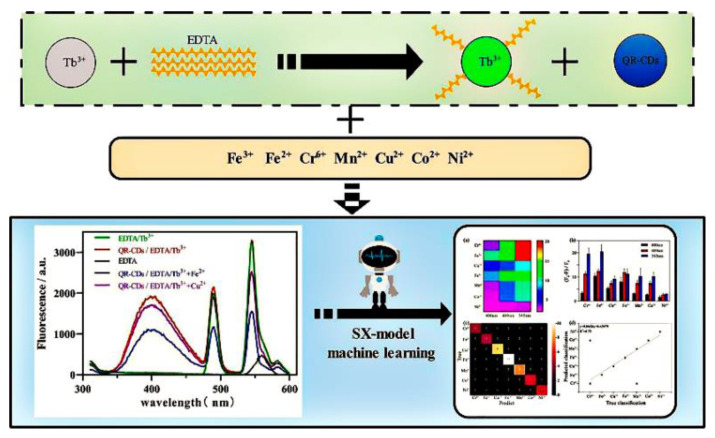
Schematic diagram of the multi-emission array sensor used to build and detect heavy metal ions. Reprinted with permission from [78]. Copyright (2022) Elsevier.

Fluorescence array-based sensing technology is developing as an emerging platform that allows the sensitive detection of analytes in complex environments, as it does not require specific recognition effects. The sensor array can generate a differential fingerprint pattern, which is combined with machine learning algorithms and enables analyte detection with improved sensitivity. Pan et al. constructed a fluorescence-array-based sensor using CDs with different surface functions to successfully distinguish eight different trace proteins. Machine learning algorithms are also used to process the fluorescent signals from the array more efficiently [77]. Shi and his colleagues constructed a multi-emission fluorescence sensor array from CDs and lanthanide complexes (EDTA-Tb^3+^), which was capable of obtaining multi-dimensional data for multiple heavy metal ions simultaneously (Figure 13). Based on the response between the metal ions and the fluorescence sensor, the fluorescence analysis maps were created for each metal ion. The multi-dimensional datas obtained from the array were analyzed using machine learning to achieve the differentiation of multiple heavy metal ions. Additionally, the accuracy rates for characterizing the metal ions in lake water and soil were 93.3% and 100%, respectively [78].

**Figure 14:**
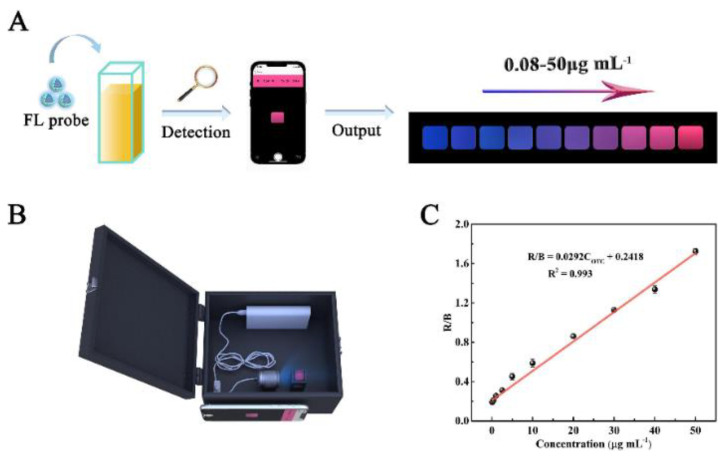
(**A**) Schematic diagram of using the developed smartphone-integrated sensing platform to detect OTC. (**B**) The render 3D views of homemade device. (**C**) Linear relationship between R/B of the portable sensing platform and the OTC concentrations (OTC concentrations: 0.08, 0.4, 1, 2.5, 5, 10, 20, 30, 40, 50 μg/mL). Reprinted with permission from [62]. Copyright (2022) Elsevier.

In recent years, emerging technologies based on the integration of intelligent devices and point-of-care testing platforms have met the food testing requirements as well as the need for rapid analysis. Meanwhile, smartphone-based ratiometric fluorescence analyses of food contaminants have been rapidly developed by combining the advantages of ratiometric fluorescence strategies and visual analyses using smartphones [3]. Wang et al. constructed Mg,N-CDs/Eu-MOFs@MIP with dual emissions by encapsulating Mg,N-CDs in Eu-MOFs via a facile self-assembly strategy combined with molecular blotting (Figure 14). Based on the color change of the system, they achieved the visual sensing of OTC via a color analysis of fluorescent images using a smartphone as a signal reader and analyzer. To demonstrate the practical applicability of the developed smartphone-based test platform, the device was utilized for the on-site detection of OTC in water and milk samples [62].

Xiao et al. prepared a smartphone detection kit with hydrogel-immobilized CD-CdTe QDs, which enabled the on-site detection of dichlorvos in water or food. The emission of CDs was not responsive to Cu^2+^, whereas the fluorescence of CdTe QDs was quenched through electron transfer by Cu^2+^. The CDs and CdTe QDs were embedded in the hydrogel, thereby preparing a ratiometric fluorescence probe of CDs and CdTe QDs. The acetylcholinesterase hydrolyzed acetylthiocholine to produce thiocholine, which binds tightly to Cu^2+^. However, dichlorvos was shown to inhibit the activity of acetylcholinesterase, thereby affecting the ratiometric fluorescence signal. A smartphone with a built-in camera was installed to capture the changes in test kit emission colors, which were converted to green and red channel intensities using ImageJ software. The sensitive detection of dichlorvos was achieved via the relation between the intensity ratio of green and red channels and the concentration of dichlorvos. The portable assay kit can be completed in 50 min with a detection limit down to 0.38 ppb [79].

## 6. Summary and Outlook

Nowadays, the issue of food safety has risen to the top of the list of pressing global concerns. It is essential to use a rational research technique in order to confirm a food’s quality. In this review, we emphasize the current developments in RFS based on CDs for foodborne contaminant detection in the past five years, focusing on surface modifications of CDs, fluorescence sensing mechanisms, and RFS strategies.

As we all know, the intrinsic emission strength of bare CDs is weak. To improve the detection sensitivity, modification by functionalization is a good strategy. The functionalized modifications include heteroatom doping and surface modification, which can change the physical and optical properties by effectively tuning the intrinsic structure and surface states of the CDs. The functionalization strategy allows the CDs to show great potential for use in biosensors, fuel cells, and photocatalytic processes. Due to the inadequate understanding of the CDs’ structure, it is still highly hard to comprehend how surface modifications alter the intrinsic structure and luminescence mechanism. Currently, the primary response mechanisms for foodborne contaminant detection based on CDs include the SQE, FRET, IFE, and the AIE. CDs with a multitude of surface functional groups can improve the selectivity of the detection process by further modifying specific recognition units such as aptamers, MIPs, and antibodies. Meanwhile, to overcome the interference of various components in complex food matrices, the use of ratiometric fluorescence based on CDs has attracted much attention because of the unique self-calibration properties.

In foodborne contaminant detection, ratiometric fluorescence probes based on CDs or in combination with other nanomaterials can be categorized into CDs as dual-emitting substrates, reference signals, and response signals. Dual-emission CDs are biocompatible and simple to prepare, making them a new class of RFS. Meanwhile, CDs can also be coupled with QDs, MNCs, rare earth metals, and MOFs to construct ratiometric fluorescence probes based on nanohybrid CDs. Therefore, the development of ratiometric CD fluorescence sensors is still challenging. The emission behavior and detection mechanism of CDs with dual-emission properties are still not well understood, which also limit their development. Meanwhile, the combination of CDs with other fluorophores to construct RFS may introduce toxic, hydrophobic groups, and may also affect the fluorescence performance of the CDs. Furthermore, the current focus in foodborne contaminant detection are on a few common analytes such as TC or organophosphorus pesticides, while the analytical methods for other analytes are still limited. The detection of analytes in different food matrices requires different RFS for CDs, which limits their application. We believe that future research studies should actively explore new methods based on CDs for the multiplexing and high-throughput detection of foodborne contaminants.

Smartphone-based analysis methods, with their portability, programmability, and visualization ability, offer new opportunities for foodborne contaminant detection. The conjunction of smartphone software programs and ratiometric fluorescence strategies has revealed a wide range of usage options within the scope of food safety detection. However, complex matrices of food products need to be pretreated in the assay. Hence, there is a great need to develop automated sample pretreatment microsystems that are compatible with smartphones. Meanwhile, the multiplexing of food pollutants has been made possible by the invention of ratiometric fluorescent signal-driven smartphone sensors. In conclusion, we believe that portable smartphone technologies based on CD probes have a promising future in foodborne contaminant detection. Their commercialization is imminent and the topic will continue to advance in future research. At the same time, the establishment of new methods for joint detection in the fields of nanomaterials, optics, and intelligent science will continue to improve the research on foodborne contaminants.

## Figures and Tables

**Table 2:** Summary of fluorescence sensors with ratiometric CDs as response signals.

Fluorescent Probes	Analytes	Detection Mechanism	LOD	Ref.
rQDs@SiO_2_@CDs	Fomesin	Fomesin induced AuNPs aggregation	59 nM	[54]
QDs@SiO_2_@CDs	Hg^2+^	SQE	0.47 nM	[55]
CDs/CuNCs	DA	Electronic transfer	32 nM	[56]
N-CQDs/AuNCs	Polymyxin	FRET	0.83 μM	[57]
CDs/AgNCs	ochratoxin A	FRET	8.7 nM	[58]
(Gd,Eu)_2_O_3_-PEI@CD	Curcumin	IFE	0.0615 μg/mL	[59]
N-CDs-Tb-DPA	Hg^2+^	Electronic transfer	37 nM	[60]
Eu/CMP-cit CPNs	TC	IFE/AE	8 nM	[61]
Mg,N-CDs/Eu-MOFs@MIP	Tonomycin	IFE/AE	6.6 ng/mL	[62]
CDs-RhB	nitrite	Griess-like mechanism	67 nM	[63]
CDs/riboflavin	CIP	Hydrogen bonding/Conjugation effect	0.13 μM	[64]
CMH-GA-CDs	Cu^2+^	FRET	0.21 μM	[65]
CDs/DA	OPs	PET	0.025 pg/mL	[66]
S,N-CDs/TC	TC	IFE	0.25 μM	[67]

## Data Availability

Not applicable.

## Abbreviations

AE	Antenna effect
AgNPs	Silver nanoparticles
AIE	Aggregation-induced luminescence
ALP	Alkaline phosphatase
Asp	Aspartic acid
AuNCs	Gold nanoclusters
BAs	Biogenic amines
B-CDs	Blue fluorescent CDs
B-CQD	Boron-doped CQD
CDs	Carbon dots
CQDs	Carbon quantum dots
CTC	Chlortetracycline
CMH	7-diethylamino coumarin-3-carbohydrazide
CMP	Cytidine monophosphate
DA	Dopamine
DPA	Dipicolinic acid
Eu-CDs	Europium-doped CDs
FLQY	Fluorescence quantum yield
FRET	Fluorescence resonance energy transfer
GA-CDs	Glyoxylic-acid-modified CDs
GQDs	Graphene quantum dots
IFE	Internal filtration effect
LC	λ-cyfluthrin
L-Cys	L-cysteine
MNA	6-Mercaptonicotinic acid
MNCs	Noble metal nanoclusters
MOFs	Metal-organic frameworks
MIP	Molecular Imprinting Polymer
MUA	11-Mercaptoundecanoic acid
NCDs	Nitrogen-doped CDs
NP	Nitrobenzyl phenol
OPD	O-phenylenediamine
OPs	Organophosphorus pesticides
OTC	Oxytetracycline
RCDs	Red fluorescent CDs
RFS	Ratiometric fluorescence sensors
RhB	Rhodamine B
SQE	Static quenching effect
TC	Tetracycline
μPAD	Microfluidic paper-based analytical devices
Y-CDs	Yellow fluorescent CDs
